# Transcriptome-wide co-expression analysis identifies *LRRC2* as a novel mediator of mitochondrial and cardiac function

**DOI:** 10.1371/journal.pone.0170458

**Published:** 2017-02-03

**Authors:** Chris McDermott-Roe, Marion Leleu, Glenn C. Rowe, Oleg Palygin, John D. Bukowy, Judy Kuo, Monika Rech, Steffie Hermans-Beijnsberger, Sebastian Schaefer, Eleonora Adami, Esther E. Creemers, Matthias Heinig, Blanche Schroen, Zoltan Arany, Enrico Petretto, Aron M. Geurts

**Affiliations:** 1 Cardiovascular Center, Medical College of Wisconsin, Milwaukee, WI, United States of America; 2 School of Life Sciences, Ecole Polytechnique Fédérale de Lausanne (EPFL), Lausanne, Switzerland; 3 Division of Cardiovascular Disease, University of Alabama at Birmingham, Birmingham, AL, United States of America; 4 Center for Heart Failure Research, Department of Cardiology, CARIM School for Cardiovascular Diseases, Maastricht University, Maastricht, The Netherlands; 5 National Heart Research Institute Singapore, National Heart Centre Singapore, Singapore, Singapore; 6 Cardiovascular and Metabolic Sciences, Max Delbrück Center for Molecular Medicine, Berlin, Germany; 7 Experimental Cardiology, Academic Medical Center, University of Amsterdam, Amsterdam, The Netherlands; 8 Institute of Computational Biology, Helmholtz Zentrum München, Neuherberg, Germany; 9 Cardiovascular Institute, Perelman School of Medicine, University of Pennsylvania, Philadelphia, Pennsylvania, United States of America; 10 MRC Clinical Sciences Centre, Imperial College London, London, UK, Duke-NUS Graduate Medical School, Singapore, Singapore; Hospital Universitari i Politecnic La Fe, SPAIN

## Abstract

Mitochondrial dysfunction contributes to myriad monogenic and complex pathologies. To understand the underlying mechanisms, it is essential to define the full complement of proteins that modulate mitochondrial function. To identify such proteins, we performed a meta-analysis of publicly available gene expression data. Gene co-expression analysis of a large and heterogeneous compendium of microarray data nominated a sub-population of transcripts that whilst highly correlated with known mitochondrial protein-encoding transcripts (MPETs), are not themselves recognized as generating proteins either localized to the mitochondrion or pertinent to functions therein. To focus the analysis on a medically-important condition with a strong yet incompletely understood mitochondrial component, candidates were cross-referenced with an MPET-enriched module independently generated via genome-wide co-expression network analysis of a human heart failure gene expression dataset. The strongest uncharacterized candidate in the analysis was *Leucine Rich Repeat Containing 2* (*LRRC2*). LRRC2 was found to be localized to the mitochondria in human cells and transcriptionally-regulated by the mitochondrial master regulator Pgc-1α. We report that *Lrrc2* transcript abundance correlates with that of *β-MHC*, a canonical marker of cardiac hypertrophy in humans and experimentally demonstrated an elevation in *Lrrc2* transcript in *in vitro* and *in vivo* rodent models of cardiac hypertrophy as well as in patients with dilated cardiomyopathy. RNAi-mediated *Lrrc2* knockdown in a rat-derived cardiomyocyte cell line resulted in enhanced expression of canonical hypertrophic biomarkers as well as increased mitochondrial mass in the context of increased *Pgc-1α* expression. In conclusion, our meta-analysis represents a simple yet powerful springboard for the nomination of putative mitochondrially-pertinent proteins relevant to cardiac function and enabled the identification of *LRRC2* as a novel mitochondrially-relevant protein and regulator of the hypertrophic response.

## Introduction

Mitochondria are highly abundant organelles, found in almost every eukaryotic cell, and are best known for production of adenosine triphosphate via oxidative phosphorylation. However, the functional remit of the mitochondrion extends far beyond this canonical role as the ‘powerhouse of the cell’ [1]. Mitochondria regulate multiple essential facets of cellular metabolism and physiology including synthesis, breakdown and interconversion of amino acids, maintenance and modulation of ion gradients, and regulation of pyrimidine and heme biosynthesis [2]. Given the diverse and essential nature of such processes, it follows that perturbations therein result in varied and often severe phenotypic manifestations. Mitochondrial diseases (MD) aggregately represent the most prevalent cause of inborn errors of metabolism and encompass a clinically heterogenous group of multisystemic disorders—with symptoms including myopathy, encephalopathy, lactic acidosis, neuropathy, liver failure, ataxia, deafness and optic atrophy—that result from defective mitochondrial function [3]. Mitochondrial dysfunction is also a feature of many common diseases including Alzheimer’s [4], Parkinson’s [5], hypertrophic cardiomyopathy [6], and cancer [7]. Elucidating how mitochondrial dysfunction contributes to disease states, either as the principal cause of monogenic disorders or as a compounding feature in complex disease, will undoubtedly augment diagnostic success and therapeutic prospects. The stochastic nature of mitochondrial disease (e.g., multiple modes of inheritance, variable age of onset and wide symptom spectrum [8] and challenges associated with reverse-genetics approaches means functional genomics, proteomics and forward genetics-based strategies have played an important role in the discovery of disease-associated genes. Capturing the full complement of mitochondrial proteins has been the focus of much effort for the last two decades [9] and mass spectrometry-based methods have been especially useful in the shaping understanding of the mitochondrial proteome [10, 11]. MitoCarta is a collection of approximately 1,100 proteins with experimental evidence of mitochondrial localization and is the most expansive self-contained survey to date [10]. As well as highlighting the functional heterogeneity of the organelle, MitoCarta has enabled the identification of multiple disease-causing genes [12–16]. In addition to the proteins reported in the MitoCarta database and companion studies [17, 18], it is estimated that a substantial number, perhaps several hundred, remain unassigned and efforts to fill this gap continue.

Left ventricular hypertrophy refers to a cardiac growth response triggered by increased mechanical stress (e.g., pressure overload, valvular disease and hypertrophic cardiomyopathy) and represents a major risk factor for heart failure and all-cause mortality [19]. Mitochondrial activity impinges profoundly on cardiomyocyte function, which in turn modulates organ-level physiology and performance. For example, angiotensin II-mediated production of reactive oxygen species in cardiomyocytes occurs primarily in the mitochondrion and leads to a hypertrophic growth response [20] while deficiency of TFAM, a key regulator of mitochondrial DNA maintenance and transcription, triggers a fuel preference switch from fatty acids to glucose, reduces energetic competence, and eventuates in severe cardiomyopathy [21]. Additionally, the adverse effects of certain environmental toxins (e.g., paraquat) and therapeutics (e.g. doxorubicin) occur via impact on mitochondrial function [22, 23]. Left ventricular hypertrophy has a strong genetic component [24] but in contrast to more tractable physiological and metabolic traits, genetic association studies in humans have been relatively ineffective in identifying loci that modulate heart size and heart failure susceptibility. Hence, identification of the proteins and pathways that modulate heart mass and function has thus far drawn heavily on integrative genomics- and forward genetics-based approaches in rodents [25–27].

In the present study, we used a co-expression-based strategy to nominate proteins of significance to both mitochondrial and cardiomyocyte function. The premise of co-expression profiling is that transcripts that produce functionally-related proteins are often subject to common regulatory mechanisms and thus display concordant expression profiles across different samples [28]. Such *guilt-by-association* methods have previously been applied to identify mitochondrial proteins [10–12] although most studies used relatively small datasets and most genes identified exhibited readily obvious co-expression metrics. We posited that the burgeoning quantity of mineable large-scale datasets readily obtainable from databases such as Gene Expression Omnibus and ArrayExpress would permit identification of proteins with less overt co-expression patterns or those whose transcripts were not interrogated via the array formats used in previous studies. Using a two-phase transcriptional correlation meta-survey that involved a large-scale mitochondrion-centric multi-tissue screen followed by a cardiac-focused analysis, we generated a novel hypothesis regarding *LRRC2*, which was then experimentally-confirmed as a novel mitochondrially-associated protein and mediator of the hypertrophic response in cardiomyocytes. Mechanistically, our findings are suggestive of a role for LRRC2 as a dampener of mitochondrial biogenesis and a positive regulator of cardiac hypertrophy via coordinated modulation of PGC-1α activity. This study further underscores the utility of unbiased and transcriptome-wide methods for the nomination of disease-relevant proteins and assigns a mitochondrially-associated function to a protein for which very little was previously known.

## Results

### Gene co-expression analysis nominates a population of transcripts of potential importance in mitochondrial function

**Fig 1:**
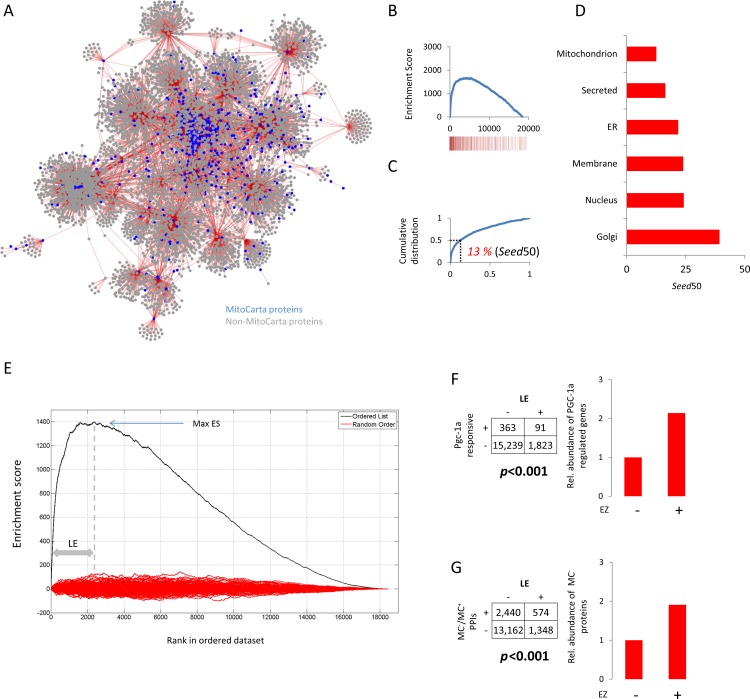
Construction of a mitochondrion-centric multi-tissue gene co-expression network. A, 935 bona fide MPETs [10] were systematically queried using the CO-Regulation Database (CORD) to identify transcripts with similar expression signatures. The resulting correlate lists were merged to create a network where nodes denote transcripts and edges join transcripts with concordant expression signatures. For any node, the extent of centrality in the network correlates with increasing connectivity. For ease of viewing, only transcripts that correlate at R≥0.8 are displayed. B, Individual correlate lists derived from CORD were concatenated to generate a ranked master list where rank is based on the number of times a given transcript was present across all daughter lists. A running-sum (Enrichment Score) was then generated by descending the list and assigning a positive score (up-step) when a transcript was a seed and a negative score (down-step) when it was not. The barcode denotes positions in ranked list of the seed population. C, Cumulative distribution analysis of the ranked master list from B to quantify the extent of network intra-correlation, expressed as the percentage of the list containing half the seed population, reveals half of the seeds used to build the network were present in the top 13% of the ranked transcript list (*seed50* = 13%). D, Comparison of *seed50* values across all six major cell compartments following CORD-based network analysis. E, Data from B (mitochondrion) showing the maximum Enrichment Score (Max ES, ~1,400) and resulting leading edge (LE). The red lines denote the ESs calculated following 1000 random walks. F, Distribution of Pgc-1α-responsive transcripts within and beyond the LE sub-set. Matrix displays the number of transcripts within (+) and outside (-) the LE, regulated (+) or not (-) by Pgc-1α and the bar chart displays relative abundance. G, Distribution of proteins not documented as being mitochondrially-localised but which purportedly interact with known mitochondrial proteins, according to publicly accessible proteomics data, within and beyond the LE sub-set. Matrix displays the number of proteins within (+) and outside (-) the LE, purported to interact with known mitochondrial proteins based on analysis of BioGRID protein-protein interaction (PPI) data [40] and the bar chart displays relative abundance. MC^+^ and MC^-^ denote MitoCarta and non-MitoCarta proteins, respectively. For example, there were 574 transcripts in the LE whose protein products have not been documented to produce mitochondrial proteins but which have been reported to interact with known mitochondrial proteins. The *P* values in F and G correspond to the result of a Chi-squared test and a Fisher Exact Test (FET), respectively.

A mitochondrion-centric co-expression network was generated by searching the CO-Regulation Database (CORD) [29] for transcripts with expression signatures similar to those of bona fide mitochondrial protein-encoding transcripts (MPETs) (Fig 1A). A total of 935 MPETs (S1 Table), which we refer to herein as ‘seeds’ since they were used to create the network, were queried in this way. The network comprised ~18,000 unique transcripts and ~183,000 transcript-transcript interactions (at R>0.5) (S2 Table). For comparative value, co-expression networks for the nucleus, endoplasmic reticulum, golgi apparatus, and plasma membrane were also generated using compartment-specific seed lists (S1A Fig and S1 Table). The extent of intra-correlation inherent to each network was measured using an approach analogous to gene set enrichment analysis (GSEA) [30]. For a given compartment, all network transcripts were ordered by the number of correlations shared with other transcripts in the network and a running sum was generated by descending the ordered list and assigning up-step and down-step scores to seed and non-seed transcripts, respectively (see Methods). In each compartment, many of the transcripts that shared most correlations were seeds as evident from the skewed gene set enrichment profiles (Fig 1B and S1A Fig), supporting the notion that the expression of spatially-congruent, functionally-related transcripts is coordinately-regulated. Using the same ordered lists, cumulative distribution analyses was conducted and revealed that the mitochondrial population, with half its seed population in the top 13% of its rank list, compared with 24% (nucleus), 31% (plasma membrane), 35% (endoplasmic reticulum), and 38% (golgi apparatus), to be the most intra-correlated (Fig 1C and 1D and S1B Fig). The mitochondrial data was next explored in more depth. Given that ~1,500 of the estimated 23,000 protein-coding genes in the human genome produce mitochondrial proteins [31], we posited that chance alone would result in an approximate 7% (1,500/23,000) representation of MPETs in any given seed’s correlate list. By comparing this *by chance* value to the actual quantities, we found that 762 (>80%) of the 935 mitochondrial seeds correlate with a greater number of MPETs than would be expected by chance alone (FET *P*<0.0001) (S2A Fig). Of note, nuclear-encoded mitochondrial ribosomal protein-encoding transcripts featured prominently amongst the most highly correlated seeds (S2B Fig and S2 Table) in keeping with the phenomenon that network hub genes tend to encode proteins with transcriptional and/or translational properties due to their ‘control’ over other network genes [32, 33]. It also suggests a synchronicity between the nuclear and mitochondrial genomes and as noted elsewhere, that regulation of gene expression from the latter is modulated more via altered translational flux than transcriptional [34, 35]. Next, a high-priority sub-population was nominated for further examination. From the enrichment analysis, we defined a leading edge (LE) sub-set of transcripts (see Methods), the majority of which have not previously been linked to mitochondrial function (Fig 1E and S3 Table). Given the high degree of intra-correlation and hence MPET representation in the LE, it follows that it should be enriched with transcripts whose expression levels are modulated by Peroxisome proliferator-activated receptor gamma coactivator-1alpha (Pgc-1α), widely recognized as the master regulator of mitochondrial function [36–38]. By cross-referencing the LE with an independently-derived list of Pgc-1α-responsive genes [39], we found, as anticipated, that the LE contained relatively more transcripts regulated by Pgc-1α (results not shown). Crucially, this enrichment persisted following removal of the seed sub-population (Fig 1F and S4 Table) (Chi-square *P*<0.001). Next, we reasoned–given the physical compartmentalization afforded by the mitochondrion–that if the LE is indeed enriched for novel MPETs, then the cognate proteins would be inclined to interact with known mitochondrial proteins rather than non-mitochondrial proteins. When all ~750,000 protein-protein interactions from the BioGrid database [40] were recovered and filtered as to retain only those interaction events (20,955) in which one of the two proteins was a MitoCarta protein (we define these interactions as MC^+^/MC^-^ where MC^+^ and MC^-^ denote MitoCarta and non-MitoCarta proteins, respectively), we found preferential residence of the 5,616 MC^-^ proteins in the LE population (FET *P*<0.001) (Fig 1G and S5 Table). For added stringency, we imposed a minimal number of interactions threshold and found that when MC^-^ proteins that purportedly bind >1, >2, >5, or >10 bona fide mitochondrial proteins were selectively retained, the representation of these MC^-^ proteins in the LE relative to outside the LE became increasingly pronounced (S3A and S3B Fig and S6 Table). In summary, our meta-analysis revealed that transcripts that generate mitochondrial proteins are more highly co-regulated than transcripts that give rise to proteins destined for any other major cellular compartment. Moreover, the prioritized transcript population derived from the mitochondria network was, even upon removal of known MPETs, enriched for transcripts regulated by Pgc-1α and whose protein products interact with mitochondrial proteins, suggesting the presence of overlooked mitochondrially-relevant proteins.

### A heart failure-specific gene co-expression network nominates LRRC2 as a mediator of mitochondrial and cardiac function

**Fig 2:**
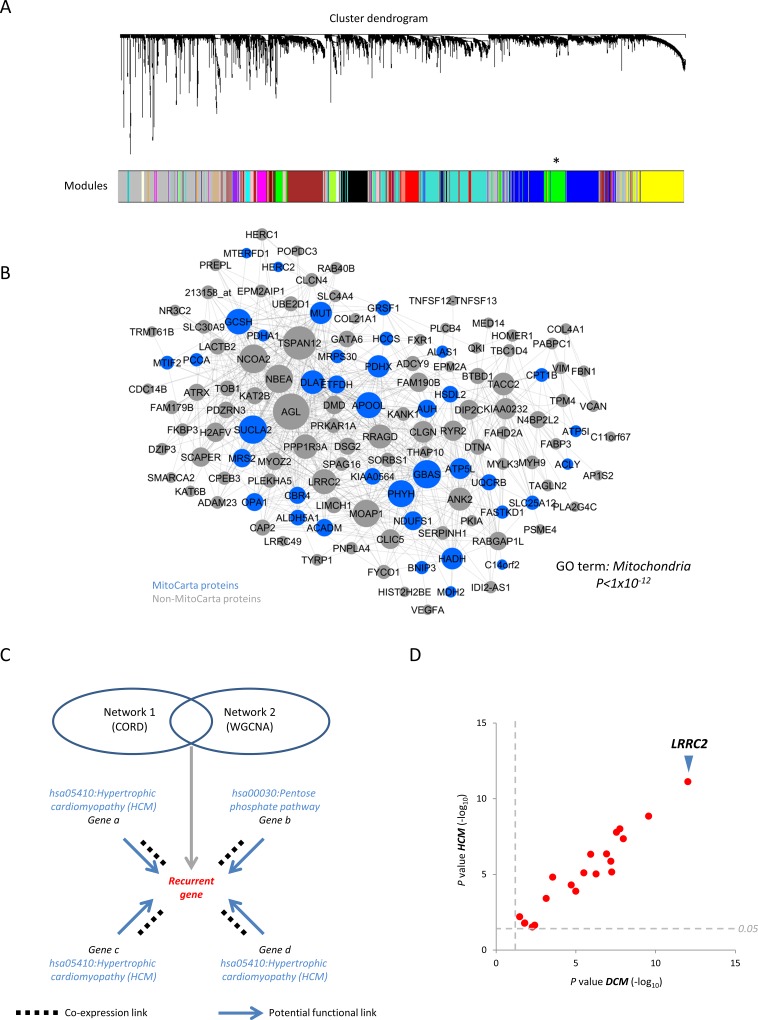
Weighted gene co-expression network analysis (WGCNA) of a human cardiac gene expression dataset and nomination of proteins potentially involved in mitochondrial and cardiac function. A, Dendrogram representation of the 27 transcript modules derived from WGCNA of a human heart failure microarray dataset (comprising healthy (n = 16), ischemic heart failure (n = 86) and idiopathic heart failure (n = 108) samples). Horizontal lines (branches) represent modules and vertical lines (leaves) represent transcripts. Asterisk denotes an MPET-enriched (green) module. B, Green module from A where transcripts are represented as nodes (size denotes matrix membership (see Methods)) and edges denote significant transcript-transcript correlations. C, Schema illustrating candidate selection following CORD and WGCNA studies. Genes nominated by both analyses were re-processed with CORD to recover co-expressed genes and KEGG terms associated with those genes. In this illustrative example, all genes that correlate with the *Recurrent gene* apart from *Gene b* are linked to Hypertrophic Cardiomyopathy suggesting that the *Recurrent gene* may also be functionally linked. D, Scatter plot showing the sub-set of recurrent genes (24 of 78) that correlate with a disproportionately large number of genes linked to Hypertrophic Cardiomyopathy (HCM) and Dilated Cardiomyopathy (DCM). X and Y axes denote the EASE scores (a measure of ontological enrichment [44]), expressed as -log_10_ *P* values, corresponding to DCM and HCM KEGG terms, respectively. Grey dashed lines denote the *P*≥0.05 EASE threshold.

Left ventricular hypertrophy is a risk factor for heart failure and all-cause mortality and can result from inappropriate signaling and metabolic derangement at the level of the mitochondrion [36]. Hence, we next attempted to leverage our data for the nomination of genes involved in cardiac remodeling in a mitochondrial manner. Weighted gene co-expression network analysis (WGCNA) [41] was applied to a large publicly available microarray dataset (GSE5406) derived from healthy, ischemic heart failure, and idiopathic human heart failure sample [42]. We identified an MPET-enriched module which was present in all sample sub-group networks (based on GO term enrichment (Mitochondria *P*<1x10^-12^) but strongest in a network produced by concurrently processing all sub-groups (Fig 2A and 2B and S4 Fig). Cross-reference of the prioritized population from the first CORD-based meta-analysis with the MPET module described above identified 78 common transcripts. Given functionally-related genes are often co-expressed [43], it was hypothesised that ontological descriptors associated with transcripts that correlate with these recurrent candidates would represent a useful proxy for indirectly inferring functionality (Fig 2C). Hence, transcripts that correlated with each candidate were recovered using CORD and then probed for over-representation of pertinent KEGG terms (dilated cardiomyopathy (DCM) and hypertrophic cardiomyopathy (HCM)). Twenty-four of the recurrent transcripts possessed such enrichment and Leucine Rich Repeat Containing 2 (*LRRC2*) exhibited the most significant guilt-by-association with both diseases (HCM *P*<10x10^-14^; DCM *P*<8x10^-12^) (Fig 2D).

### LRRC2 is mitochondrially-localized, transcriptionally-regulated by Pgc-1a, and regulates mitochondrial biogenesis

**Fig 3:**
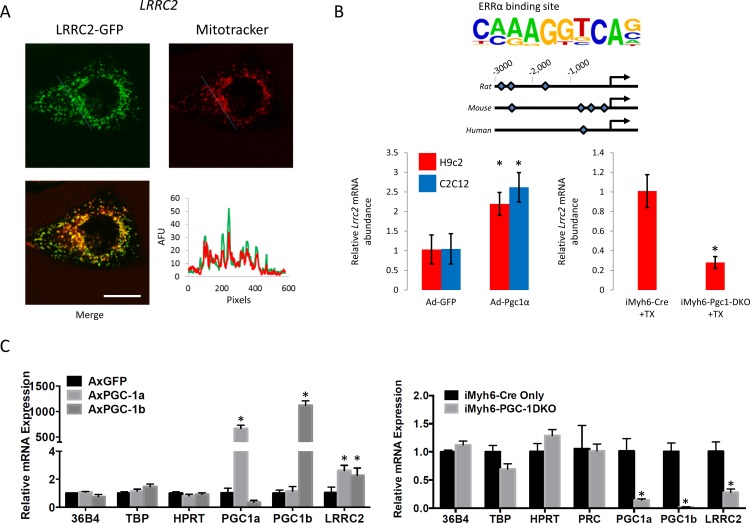
LRRC2 localizes to the mitochondria and is regulated by Pgc-1. A, HEK293 cells expressing a LRRC2-GFP fusion protein were incubated with MitoTracker Red and then imaged via confocal microscopy (left). Chart displays signal co-occurrence (green, LRRC2; red, MitoTracker Red) along the dashed line in unmerged images. Scale bar = 20μm. B, Approximate positions of potential ERRα binding sites in rat, mouse and human *LRRC2* genes (top panel), *Lrrc2* mRNA abundance in C2C12 and H9c2 cells following infection with either a GFP- or Pgc-1α-encoding adenovirus (bottom-left panel), and *Lrrc2* mRNA abundance in the cardiac-specific inducible Pgc-1α/β double-knockout (DKO) mouse 3 weeks post-tamoxifen (TX) treatment (bottom-right panel). C, Transcript abundance of *Lrrc2* and control transcripts (*36b4*, *Tbp* and *Hprt*) as well as Pgc-1α/β in C2C12 cells following adenovirus-mediated over-expression of Pgc-1α and Pgc-1α/β. D, Transcript abundance of *Lrrc2* and control transcripts (*36b4*, *Tbp* and *Hprt*) as well as Pgc-1α/β in hearts of cardiac-specific Pgc-1α/β double knockout (DKO) mice and Cre only control mice. All QPCR data are represented as means ± s.e.m and are derived from three independent experiments. *, *P*≤0.05.

How, if at all, *LRRC2* may interface with mitochondrial and cardiac function is unknown and despite belonging to one of the most evolutionarily-conserved and functionally-important protein families [45], remains understudied. A LRRC2-GFP chimeric protein was expressed in HEK293 cells and was observed to associate with the mitochondria and to a lesser extent the cytosol and nucleus (Fig 3A). *In silico* profiling of a 3,000bp sequence upstream of the transcription start site in the human, rat and mouse gene revealed multiple Estrogen-related receptor alpha (ERRα) binding sites (Fig 3B, top panel), suggestive of regulation by Pgc-1α and thus a role in fundamental mitochondrial function. We experimentally assessed this finding by over-expressing Pgc-1α in mouse C2C12 cells and rat H9c2 cells via adenovirus-mediated gene transfer and found that doing so invoked upregulation of the *Lrrc2* transcript (Fig 3B, bottom left and Fig 3C). Pgc-1-mediated regulation of *Lrrc2* was also explored *in vivo* using a cardiac-specific inducible Pgc-1α/β double knockout mouse. Pgc-1α/β loss led to a marked reduction in *Lrrc2* transcript abundance (Fig 3B, bottom right panel and Fig 3D).

**Fig 4:**
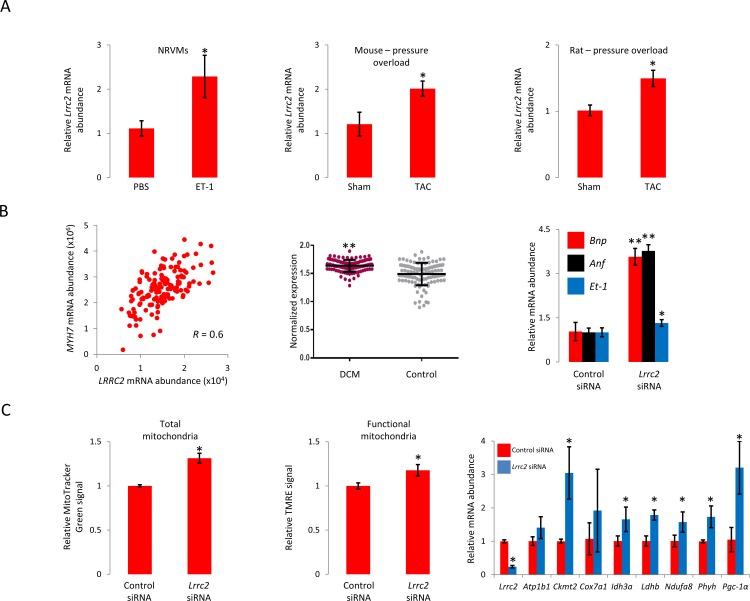
LRRC2 modulates mitochondrial and cardiomyocyte function. A, *Lrrc2* mRNA abundance in PBS- and ET-1-treated neonatal rat ventricular myocytes (left panel), and left ventricle of mouse (middle panel) and rat (right panel) 4 weeks post-aortic banding compared to sham-operated controls. B, Correlation in gene expression between *LRRC2* and hypertrophic biomarker *MYH7* (*β-MHC*) in the human heart (left panel), covariate-corrected *LRRC2* mRNA abundance in myocardial samples derived from dilated cardiomyopathy (DCM) patients (n = 97) and non-failing controls (n = 108) (middle panel) (*P* value calculated via Wilcoxon rank sum test), and effect of *Lrrc2* loss-of-function in H9c2 cells on expression of hypertrophic biomarkers *Bnp*, *Anf*, *and Et-1* (right panel). C, Effect of siRNA-mediated Lrrc2 loss-of-function in H9c2 cells on the abundance of total (left panel) and functional (middle panel) mitochondrial abundance, and mitochondrial protein-encoding transcript expression (right panel). All QPCR data are represented as means ± s.e.m and are derived from three independent experiments. *, *P*≤0.05; **, *P*≤0.001.

The cardiac functionality of *Lrrc2* in the context of hypertrophic remodelling was next investigated in more detail. A significant elevation in *Lrrc2* mRNA was observed in neonatal rat ventricular myocytes following treatment with endothelin-1 as well as in the myocardium of mice and rats following thoracic aortic constriction (TAC)-induced cardiac hypertrophy, indicating a potential role in maladaptive hypertrophic remodelling (Fig 4A). We also observed a correlation in the human heart (left ventricle) between *LRRC2* mRNA abundance and that of *MYH7* (R = 0.61), an established hypertrophic biomarker (Fig 4B, left panel). To address a possible link to human disease, we queried *LRRC2* expression in a left ventricle-derived RNASeq dataset derived from dilated cardiomyopathy (n = 140) and healthy control (n = 110) subjects and observed elevated *LRRC2* transcript expression in the patient cohort (Fig 4B, middle panel). In the light of these results, we postulated that variation in *Lrrc2* mRNA levels may be relevant to pathological hypertrophy–either as an initiating or buffering event—and explored the effect of loss-of-function in cardiomyocytes. Transfection of H9c2 rat cardiomyocytes with a *Lrrc2*-specific siRNA reduced mRNA abundance by >75% (data not shown) and was concomitant with induction of canonical hypertrophy marker genes atrial natriuretic peptide, brain natriuretic peptide, and endothelin-1 (Fig 4B, right panel), suggesting that Lrrc2 loss-of-function may contribute to reactivation of the fetal gene program. We next turned our attention to the mechanism through which *Lrrc2* suppression initiates the pro-growth response and asked whether the hypertrophy coincident with its loss could be due to modified mitochondrial function. No overt morphological difference in the mitochondrial network was observed following *Lrrc2* knockdown (S5 Fig) but a modest yet measurable increase in gross and functional mitochondrial mass was noted, as evident from elevated MitoTracker Green and TMRE uptake, respectively (Fig 4C, left and middle panels). Consistent with *Lrrc2* loss-of-function promoting mitochondrial biogenesis (as opposed to repressing removal of damaged or older mitochondria), abundance of Pgc-1α transcript and several Pgc-1α-regulated transcripts were elevated following RNAi-mediated Lrrc2 depletion (Fig 4C, right panel). In light of the transcriptional activation of *Lrrc2* by Pgc-1α and that repression of *Lrrc2* increased *Pgc-1α* expression, we speculated as to the existence of a Lrrc2-mediated, non-canonical feedback mechanism that may blunt the ability of *Pgc-1α* to auto-activate and hence measured *Pgc-1α* mRNA via QPCR following adenovirus (Ad)-mediated over-expression in H9c2 cells in the presence or absence of Ad-mediated LRRC2 over-expression. Ad-Pgc-1α alone induced ~2,000-fold increase in *Pgc-1α* transcript relative to Ad-GFP cells while co-infection of Ad-Pgc-1α and Ad-Lrrc2 induced a ~300-fold increase in *Pgc-1a* transcript (S6 Fig). In sum, these data suggest that Lrrc2 localizes at least partially to the mitochondria and functions to fine-tune cardiomyocyte growth through a mechanism involving Pgc-1α-dependent modulation of mitochondrial abundance.

## Discussion

The premise of co-expression analysis is that transcripts that correlate with a particular query transcript have an increased likelihood of being functionally-related to that particular query transcript. Certain gene classes are particularly well suited to co-expression-based analyses. For example, many heat shock and chaperone protein-encoding genes exhibit coordinated changes in expression following modulations in their upstream regulator *HSF1* [46]. Similarly, many nuclear genes that give rise to mitochondrial proteins are expressed in a coordinated fashion due to a relatively finite collection of upstream regulators. By way of example, Peroxisome proliferator-activated receptor gamma coactivator 1-alpha (PGC-1α) regulates the expression of 70% of the one hundred or so electron transport chain sub-unit (and ATP synthase) genes as well as all eight enzymes in the Citric Acid cycle [38]. Such synchronicity affords significant predictive power and has been exploited to identify multiple mitochondrial protein-encoding genes [10, 12]. In this study, we used a predictive methodology based on gene co-expression to nominate proteins relevant to mitochondrial function in the context of the metabolic and signaling derangement associated with pathological cardiac hypertrophy. We first conducted a lenient and inclusive correlation analysis to nominate novel mitochondrial protein-encoding transcripts (MPETs). This was accomplished by probing almost 10,000 publicly available datasets with the CORD database [29] for transcripts whose expression signatures resemble those of bona fide MPETs. In the second phase of the study, a less agnostic approach was adopted in that we asked if the phase one candidates could be rediscovered in a disease-relevant setting. To accomplish this, we created a gene network from a human heart gene expression dataset [42] via WGCNA and identified a module highly enriched in MPETs. Leucine Rich Repeat Containing 2 (*LRRC2*) was among this recurrent population and attracted interest on account of it correlating with transcripts associated with cardiac remodeling as well as a general paucity of knowledge regarding its function. We found LRRC2 to be at least partially localized to the mitochondrion and regulated by the mitochondrial master regulator PGC-1α. As well as being elevated in modeled hypertrophic settings, we also observed paralleled expression between *LRRC2* and *MYH7*, an established biomarker of heart failure, in the human heart [47]. Moreover, *Lrrc2* suppression in a cardiomyocyte cell line invoked transcriptional changes typically associated with pathological hypertrophy and a concomitant elevation in mitochondrial mass in the context of increased *Pgc-1α* expression. These data implicate a role for *LRRC2* in the control of processes that dictate cardiomyocyte hypertrophy and mitochondrial abundance. How, mechanistically, *LRRC2* loss-of-function invokes an increase in mitochondrial biogenesis is uncertain and requires further examination. In light of the association between compensated cardiac hypertrophy and Pgc-1α-driven mitochondrial biogenesis, it’s tempting to speculate that Lrrc2-mediated regulation of Pgc-1α could influence the hypertrophic response. Defining how LRRC2 fits into this model warrants additional study but one possibility, suggested by the regulatory interplay observed (i.e., that Pgc-1α overexpression and suppression leads to increased and decreased Lrrc2 expression, respectively and that Lrrc2 loss-of function elevates Pgc-1α expression) is that a protein-protein interaction between Lrrc2 and Pgc-1α may dampen the capacity of the latter to auto-regulate. Indeed, when Pgc-1α was over-expressed in H9c2 cells in combination with Lrrc2, the increase in Pgc-1α transcript abundance was far less than that observed when it was over-expressed alone. Enhanced presence of such repressive signals may contribute to the gradual reduction in mitochondrial function observed in heart failure and type 2 diabetes [48–50], and hence represents a rational target for therapeutic intervention.

As well as nominating hitherto mitochondrially-relevant proteins for the second phase of the study, the prioritized population provided corroborative insight into the regulation of mitochondrial gene expression. Ribosomal protein-encoding transcripts were by class the most highly represented when the population was reanalyzed by gene set enrichment analysis. Regulators of gene expression typically assume central, highly correlated positions in transcriptional networks because of their capacity to alter expression levels of other network genes. That mitochondrial ribosomal proteins, and not transcriptional regulators (e.g., DNA Polymerase gamma), were highly represented indicates that modulations in flux at the level of translation exert greater control over expression than transcription and are consistent with previous findings that implied pruning of the apparently surplus population of mtDNA-derived transcripts is the principal mechanism through which protein abundance is modulated.

Our nominated population of mitochondrially-associated proteins is far larger than the undiscovered mitochondrial protein population proposed by other studies. That said, extra-mitochondrial localization of a protein and its relevance to mitochondrial function are not mutually-exclusive properties [2, 3, 37, 51–53] and the shortfall in the molecular diagnosis of suspected cases of mitochondrial disease [16] is likely due in part to extra-mitochondrial proteins that influence facets of mitochondrial function. Hence, we envisage the protein population nominated in this study as representing a useful starting point for additional candidate gene- and screening-based investigations aimed at cataloging additional proteins and pathways that regulate mitochondrial function.

## Materials and methods

### Network studies

The Co-Regulation Database (CORD) [29] is a collection of over 9,000 microarray datasets which enables identification of transcripts with expression profiles similar to a user-specified query (or ‘seed’) transcript. Upon query, CORD returns a table comprising transcripts with expression signatures which correlate (based on the Pearson correlation coefficient (R)) with that of the seed. Of 1,127 known mitochondrial protein-encoding transcripts (MPETs) [10] queried in this way, 935 yielded correlate lists. (The remaining 192 were not expressed highly enough or represented in a sufficient quantity of datasets in CORD to generate correlate lists.) All seed-correlate interactions (at R≥0.5) across all 935 correlate lists were imported into and visualized using Cytoscape. To generate co-expression networks for other sub-cellular compartments (nucleus, endoplasmic reticulum, golgi apparatus, and plasma membrane), all human genes (Ensembl Human release 85) were firstly classified based on the sub-cellular compartment occupied by their protein product using gene ontology (GO) Inferred from Direct Assay (IDA) terms downloaded from Biomart. Co-expression networks for each compartment were then generated as described for the mitochondrion.

Weighted gene co-expression analysis (WGCNA) was carried out as previously described [27] on dataset GSE5406 [42] using the R package [41]. Details of all steps and functions can be found in the package guide (https://cran.r-project.org/web/packages/WGCNA/WGCNA.pdf) and code pertaining to this analysis is obtainable upon request. Briefly, all samples (nonfailing controls (n = 16), ischemic heart failure (n = 86), and idiopathic heart failure (n = 108)) were RMA-normalized and assessed for outliers and missing data using the goodSamplesGenes function. Gene modules were constructed using the blockwiseModules function with a soft-thresholding power = 9 and the significance of module assignment for each gene was determined from Student asymptotic p-values calculated using the corPvalueStudent function. Finally, Gene Ontology analysis of each module was conducted using DAVID [54].

Transcripts co-nominated by CORD- and WGCNA-based studies were subjected to an additional correlation-based guilt-by-association analysis as an indirect means to infer involvement in pathological cardiac remodeling. This involved querying recurrent transcripts with CORD. From the resulting gene lists, KEGG term *P* values (which correspond to EASE scores [44], a measure of ontological enrichment) for dilated cardiomyopathy (hsa05414) and hypertrophic cardiomyopathy (map05410) were recovered where present. Focus was placed on transcripts that correlated with a higher than expected quantity of transcripts with the aforesaid KEGG terms (based on EASE score).

### Statistical tests

For enrichment analysis, a Kolmogorov-Smirnov test [55] was carried out following CORD analysis to calculate the degree to which seed transcripts were over-represented at the top of each compartment’s master list following concatenation of individual correlate lists. As in GSEA [30], each master list was ordered (by the number of times each transcript was observed across all individual correlate lists) and a running sum was produced by assigning an up-step score ($\frac{\sqrt{N-G}}{G}=4.32$) on encountering seed transcripts and a down-step score ($-\frac{\sqrt{G}}{N-G}=-0.23$) on encountering non-seed transcripts (*N* and *G* correspond to the total number of genes and the number of seed genes, respectively). For the mitochondrial data, 1,000 random walks were also carried out by randomly reassigning seed designations to the pre-ordered list and re-deriving the running sum. All transcripts located at or before the running sum’s maximum deviation from zero (the maximum Enrichment Score) were classified as leading edge (LE) transcripts and retained for further analysis. Cumulative distribution of seed transcripts in each compartment analysis was also conducted on the same ordered lists to define the portion of each list containing half of the seed transcripts used to generate the network. The calculated value (defined as *seed50*) enabled us to quantify the extent of intra-correlation inherent to each compartment.

Enrichment of experimentally-demonstrated Pgc-1α-responsive genes [39] and genes that give rise to proteins purported to interact with known mitochondrial proteins (predicted using BioGRID [40] in the LE population was calculated using a 2x2 contingency table in Prism 7 (GraphPad) of format Outcome 1 and 2 (columns) versus Group 1 and 2 (rows). In both analyses, Group 1 and Group 2 corresponded to LE and non-LE transcripts, respectively. To measure distribution of Pgc-1α-responsive genes, Outcome 1 and Outcome 2 corresponded to Pgc-1α-responsive genes and Pgc-1α-unresponsive genes, respectively. For analyzing the distribution of genes that give rise to proteins purported to interact with known mitochondrial proteins, Outcome 1 and Outcome 2 corresponded to the quantity of MC^-^ proteins *within* the EZ that interact with MC^+^ proteins and the quantity of MC^-^ proteins *outside* the EZ that interact with MC^+^ proteins, respectively (where MC^+^ protein denotes a MitoCarta protein and MC^-^ denotes a non-MitoCarta protein).

### Cloning strategies

A human *LRRC2* cDNA-containing plasmid (pCMV-SPORT6) was obtained from the Dharmacon Mammalian Gene Collection (MGC). The ORF was amplified (5’-TACATGCTAGCACCATGGGACATAAAGTGGTTGTCTTC-3’ and 5’-TACATGCTAGCCCAAGTTGAAGGCTAAAAGACACTTT-3’) using Kod DNA polymerase (EMD4Biosciences), digested with NheI and cloned upstream of and in-frame with the EGFP cassette in a CAG-EGFP vector using standard methods. Plasmid DNA (1μg) containing correctly orientated and sequence-verified *LRRC2* coding sequence was transfected into HEK293 cells (200,000) via electroporation. Cells were seeded in glass-bottomed 35mm cell culture dishes. Forty-eight hours post-transfection, cells were incubated for 1 hour with MitoTracker Deep Red FM (100nM) before being rinsed briefly with PBS and viewed at x40 magnification using a Nikon A1+ confocal microscope. Mitochondrial localization was determined following image capture via analysis of the overlap between GFP and MitoTracker signals.

### Animal studies

The experiments were approved by the Medical College of Wisconsin and University of Alabama at Birmingham Institutional Animal Care and Use Committee (IACUC) in accordance with the National Institutes of Health Guide for the Care and Use of Laboratory Animals. Animals were maintained on a 12 hour light/dark cycle with *ad libitum* access to standard rodent chow and water.

### Pgc-1-mediated regulation of Lrrc2

The Pgc-1α and Pgc-1β adenoviruses have been described elsewhere [56]. C2C12 and H9c2 cells were infected with the specified adenovirus at a multiplicity of infection of 10–30, and mRNA expression was measured, as described above, 72 hours later. Inducible cardiac-specific Pgc-1α/β knockout mice were generated by crossing the floxed *Pgc-1α/β* strain [57] with the tamoxifen inducible *Myh6-MerCreMer* strain [58]. Inducible cardiac-specific *Pgc-1α/β* knockout and control (*Myh6-MerCreMer*) mice were injected with tamoxifen (50mg/kg/day) daily for 5 days. Mice were sacrificed 2 weeks post-injection and hearts were harvested for RNA isolation and expression analysis. All animal experiments were performed according to procedures approved by the University of Alabama at Birmingham Institutional Animal Care and Use Committee (IACUC). Animals were euthanized with an intraperitoneal overdose of pentobarbital (200mg/kg), followed by cervical dislocation.

### siRNA-mediated Lrrc2 knockdown

H9c2 cells (100,000 cells/well) were seeded in a 12-well plate. The following day, cultures (n = 5) were transfected with either a *Lrrc2*-specific or a non-targeting scrambled control siRNA (Origene). For a given transfection, 5μl DharmaFECT1 and 95μl opti-MEM (Invitrogen) were mixed in one tube whilst 2.5μl of the *Lrrc2*-targeting or control siRNA (20μM) was mixed with 97.5μl opti-MEM in a second tube. Following a 5 minute incubation at room temperature, solutions were mixed and left at room temperature for a further 20 minutes. After the allotted time, cells received 800μl fresh media (DMEM supplemented with 10% heat-inactivated FBS and 1% penicillin/streptomycin) followed by the above transfection mixtures. The final concentration of siRNA was 50nM. Knockdown efficacy was assessed via QPCR (Forward: AGTGCACGAGTCAAGGACAG; Reverse: TTGAGGTGTGTCTGTTCCTTC) 2–3 days post-transfection and was found to be at least 75%.

### Measurement of mitochondrial content

Two days post-siRNA transfection, H9c2 cultures were either harvested for RNA isolation (see below) or incubated with either MitoTracker Green (100nM) for 1 hour or Tetramethylrhodamine Ethyl Ester Perchlorate (TMRE) (10nM) for 20 minutes, after which they were dispersed with trypsin-EDTA, washed twice and resuspended in 0.5 ml PBS/FBS (1%) and analysed using an Accuri C6 flow cytometer in the case of MitoTracker Green and an LSRII flow cytometer in the case of TMRE. Following selection of viable, singlet cell events (~10,000) from each sample, median fluorescence values of the *Lrrc2* siRNA- (n = 5) and scrambled control- (n = 5) transfected cultures were calculated and compared using FloJo.

### RNA isolation and expression analysis

Total RNA was extracted using either the RNeasy Mini kit (Qiagen) in the case of cell cultures or an adapted Trizol-based protocol in the case of tissue, as previously described [27]. For transcript analysis, total RNA (1μg) was reverse transcribed (iScript, Bio-Rad) and the resulting cDNA subjected to quantitative PCR (QPCR) using the iCycler iQ (Bio-Rad) instrument with gene-specific primers, all of which were assessed for linear efficiency and single amplicon formation. Unless otherwise stated, three independent samples were amplified in duplicate per group, and presented data is representative of three independent experiments. All data were analyzed using the 2^-ΔΔCT^ method [59].

### Gene expression analysis in human myocardial biopsies

As previously described [60], total RNA from 107 control and 96 DCM hearts was extracted using TRIzol reagent and RNA-seq libraries were created with the TruSeq RNA Sample Preparation Kit (Illumina). Barcoded cDNA fragments were sequenced on an Illumina HiSeq 2000 with 2 × 100 bp PE chemistry. Gene-level quantification was performed using htseq-count [61] and genome assembly GCRh37. Gene counts were normalized using variance-stabilization transformation (VST), part of the DESeq package [62], and log2 transformed.

### DCM transcriptome profiling

We measured transcriptome-wide RNA levels in left ventricle biopsies of end-stage DCM patients (n = 128) that were snap-frozen and stored in liquid nitrogen in a tissue bank at the Royal Brompton and Harefield Hospitals NHS Foundation Trust. The diagnosis of non-ischaemic DCM was confirmed from medical records, but more detailed clinical data is not available. We prepared non-stranded, poly(A)-selected RNA libraries for sequencing using the TruSeq RNA Sample Preparation Kit (Illumina) and sequenced on a HiSeq 2000 (Illumina) using paired-end chemistry. RNA-seq reads were aligned to the human reference genome GRCh37 using tophat version 1.4.1. Gene expression was quantified as reads per kilobase per million sequenced (RPKM) using an annotation based on Gencode version 19.

## Supporting information

S1 Fig Construction of gene co-expression networks for 5 major cellular organelles. Organelle-specific gene lists (created by classifying the entire protein-coding complement of the genome (Ensembl Human release 85) using Gene Ontology *Inferred from Direct Assay* (IDA) descriptors recovered from Biomart) were queried using CORD to generate co-expression networks and ranked master lists as described in Methods. A, Enrichment analysis of ranked master lists derived from each network. On descending each ranked list (in which transcripts are ranked by the number of times they correlate at *R*≥0.5 with other seed transcripts), up- and down-step scores were applied to seeds and non-seeds, respectively (see Methods). The barcode denotes positions in ranked list of the seed population. ES, Enrichment score. B, Cumulative distribution analysis of each compartment-specific ranked master list to quantify the extent of network intra-correlation, reflected by the *seed50* value (the proportion of the ranked list containing half the seed population). A small *seed50* value reflects high intra-correlation between the seed genes and a larger seed50 value reflects low intra-correlation.
(PDF)

S2 Fig Intra-correlation of mitochondrial network seed genes and enrichment of ribosomal protein-encoding transcripts. A, 7% (1,500 of 21,000) of the human genome comprises mitochondrial protein-encoding genes. Hence, a randomly generated gene list will not contain more than 7% mitochondrial protein-encoding genes. The scatter plot (each circle represents a seed gene) reveals that the vast majority (>80%) of the seed genes used to create the mitochondrial network (Fig 1) are co-expressed (R>0.5) with more than 7% mitochondrial protein-encoding genes. Horizontal line displays the 7% *by chance* threshold. Inset bar chart displays the percentage of seeds that correlate with >7% (+) or ≤7% (-) MPETs. B, Distribution of transcripts known to generate mitochondrial ribosomal proteins was analyzed via enrichment analysis. On descending the ranked list (in which transcripts are ranked by the number of times they correlate at *R*≥0.5 with other seed transcripts), up- and down-step scores were applied to transcripts producing ribosomal proteins and those not, respectively (see Methods). The maximum enrichment score (100) was significantly greater than that obtained from 1000 random walks (shown in red).
(PDF)

S3 Fig Prioritized transcript population is enriched for protein products that physically interact with known mitochondrial proteins. A, Percentage of transcripts within and outside the LE that generate proteins reported (in the BIOGRID database) to interact with ≥1, ≥2, ≥5, and ≥10 known mitochondrial proteins. B, Relative abundance of MC^-^ proteins (proteins that are not annotated by MitoCarta as being mitochondrially-loacalized but which are reported (in the BIOGRID database) to interact with known mitochondrial proteins) that interact with ≥1, ≥2, ≥5, and ≥10 known mitochondrial proteins in the LE compared to outside the LE.
(PDF)

S4 Fig Weighted gene co-expression network analysis (WGCNA) of individual sample groups from dataset GSE5406. Networks were constructed using the three sub-groups (control samples only, idiopathic samples only and ischemic samples only) as well as all samples simultaneously. In each case, horizontal lines (branches) represent modules and vertical lines (leaves) represent transcripts. Asterisks and accompanying *P* values denote MPET-enriched modules and Benjamini-corrected *P* values obtained via DAVID-based GO term analysis, respectively.
(PDF)

S5 Fig Mitochondrial morphology is not altered by LRRC2 loss-of-function. H9c2 cells transfected with either a control or *LRRC2*-specific siRNA were stained with Mitotracker Green and DAPI before being imaged via confocal microscopy (x100). Representative images demonstrate no overt alteration in the morphology of the mitochondrial network. Scale bar = 20μm.
(PDF)

S6 Fig LRRC2-mediated repression of *Pgc-1α* induction in H9c2 cardiomyocytes. H9c2 cells were infected with either a GFP adenovirus alone (1^st^ bar), equal amounts of a GFP and Pgc-1α adenovirus (2^nd^ bar), equal amounts of a Pgc-1α and a LRRC2 adenovirus (3^rd^ column), or equal amounts of a GFP and LRRC2 adenovirus (4^th^ bar). RNA was isolated and subjected to QPCR with Pgc-1α-specific primers capable of detecting transcript derived from the adenovirus and the endogenous gene (and HPRT primers for normalization). Data are represented as mean ± s.e.m from three independent experiments. *, *P*<0.05.
(PDF)

S1 Table Classification of human genes into 5 major sub-cellular compartments using GO IDA descriptors. (XLSX)

S2 Table Ranked master list tables derived from co-expression analysis for major sub-cellular compartments. (XLSX)

S3 Table Delineation of the leading edge (LE) transcript population in the ranked list derived from the mitochondrial network. (XLSX)

S4 Table Distribution of experimentally-proven PGC-1α-regulated transcripts within and outside the LE population. (XLSX)

S5 Table Distribution of transcripts within and outside the LE population whose protein products purportedly interact with bona fide mitochondrial proteins. (XLSX)

S6 Table Classification of proteins derived from LE transcripts based on how many bona fide mitochondrial proteins they purportedly interact with. (XLSX)
