# Supplementary material for: Transcriptome-wide co-expression analysis identifies LRRC2 as a novel mediator of mitochondrial and cardiac function
Source: PLoS One. 2017 Feb 3;12(2):e0170458. doi: 10.1371/journal.pone.0170458 (PMC5291451; doi:10.1371/journal.pone.0170458)
Supplement: S1 Fig — Organelle-specific gene lists (created by classifying the entire protein-coding complement of the genome (Ensembl Human release 85) using Gene Ontology Inferred from Direct Assay (IDA) descriptors recovered from Biomart) were queried using CORD to generate co-expression networks and ranked master lists as described in Methods. A, Enrichment analysis of ranked master lists derived from each network. On descending each ranked list (in which transcripts are ranked by the number of times they correlate at R≥0.5 with other seed transcripts), up- and down-step scores were applied to seeds and non-seeds, respectively (see Methods). The barcode denotes positions in ranked list of the seed population. ES, Enrichment score. B, Cumulative distribution analysis of each compartment-specific ranked master list to quantify the extent of network intra-correlation, reflected by the seed50 value (the proportion of the ranked list containing half the seed population). A small seed50 value reflects high intra-correlation between the seed genes and a larger seed50 value reflects low intra-correlation. (PDF) [file pone.0170458.s001.pdf]

Supplementary Figure 1

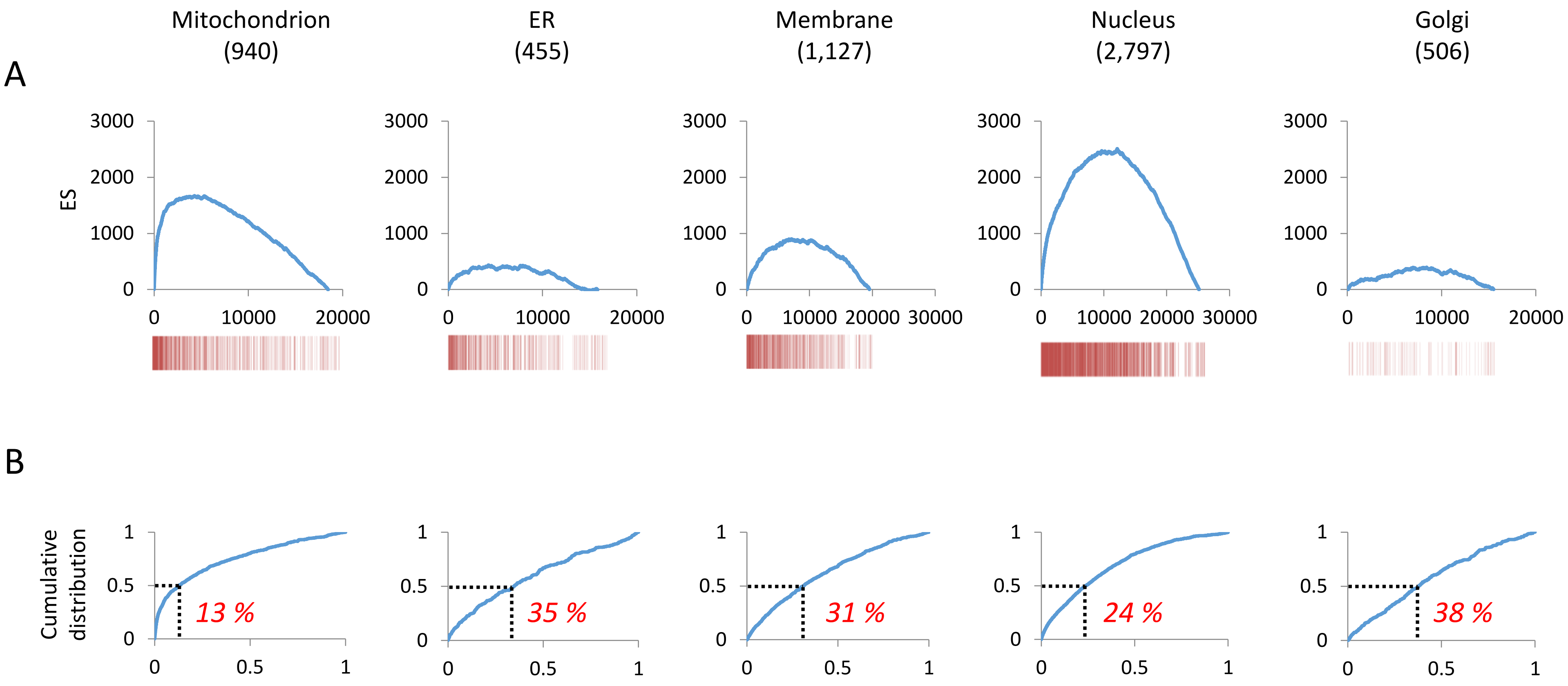

Supplementary Figure 1. Construction of gene co-expression networks for 5 major cellular organelles. Organelle-specific gene lists (created by classifying the entire protein-coding complement of the genome (Ensembl Human release 85) using Gene Ontology *Inferred from Direct Assay* (IDA) descriptors recovered from Biomart) were queried using CORD to generate co-expression networks and ranked master lists as described in Methods. A, Enrichment analysis of ranked master lists derived from each network. On descending each ranked list (in which transcripts are ranked by the number of times they correlate at $R \geq 0.5$ with other seed transcripts), up- and down-step scores were applied to seeds and non-seeds, respectively (see Methods). The barcode denotes positions in ranked list of the seed population. ES, Enrichment score. B, Cumulative distribution analysis of each compartment-specific ranked master list to quantify the extent of network intra-correlation, reflected by the *seed50* value (the proportion of the ranked list containing half the seed population). A small *seed50* value reflects high intra-correlation between the seed genes and a larger seed50 value reflects low intra-correlation.
