# Supplementary material for: Transcriptome-wide co-expression analysis identifies LRRC2 as a novel mediator of mitochondrial and cardiac function
Source: PLoS One. 2017 Feb 3;12(2):e0170458. doi: 10.1371/journal.pone.0170458 (PMC5291451; doi:10.1371/journal.pone.0170458)
Supplement: S2 Fig — A, 7% (1,500 of 21,000) of the human genome comprises mitochondrial protein-encoding genes. Hence, a randomly generated gene list will not contain more than 7% mitochondrial protein-encoding genes. The scatter plot (each circle represents a seed gene) reveals that the vast majority (>80%) of the seed genes used to create the mitochondrial network (Fig 1) are co-expressed (R>0.5) with more than 7% mitochondrial protein-encoding genes. Horizontal line displays the 7% by chance threshold. Inset bar chart displays the percentage of seeds that correlate with >7% (+) or ≤7% (-) MPETs. B, Distribution of transcripts known to generate mitochondrial ribosomal proteins was analyzed via enrichment analysis. On descending the ranked list (in which transcripts are ranked by the number of times they correlate at R≥0.5 with other seed transcripts), up- and down-step scores were applied to transcripts producing ribosomal proteins and those not, respectively (see Methods). The maximum enrichment score (100) was significantly greater than that obtained from 1000 random walks (shown in red). (PDF) [file pone.0170458.s002.pdf]

Supplementary Figure 2

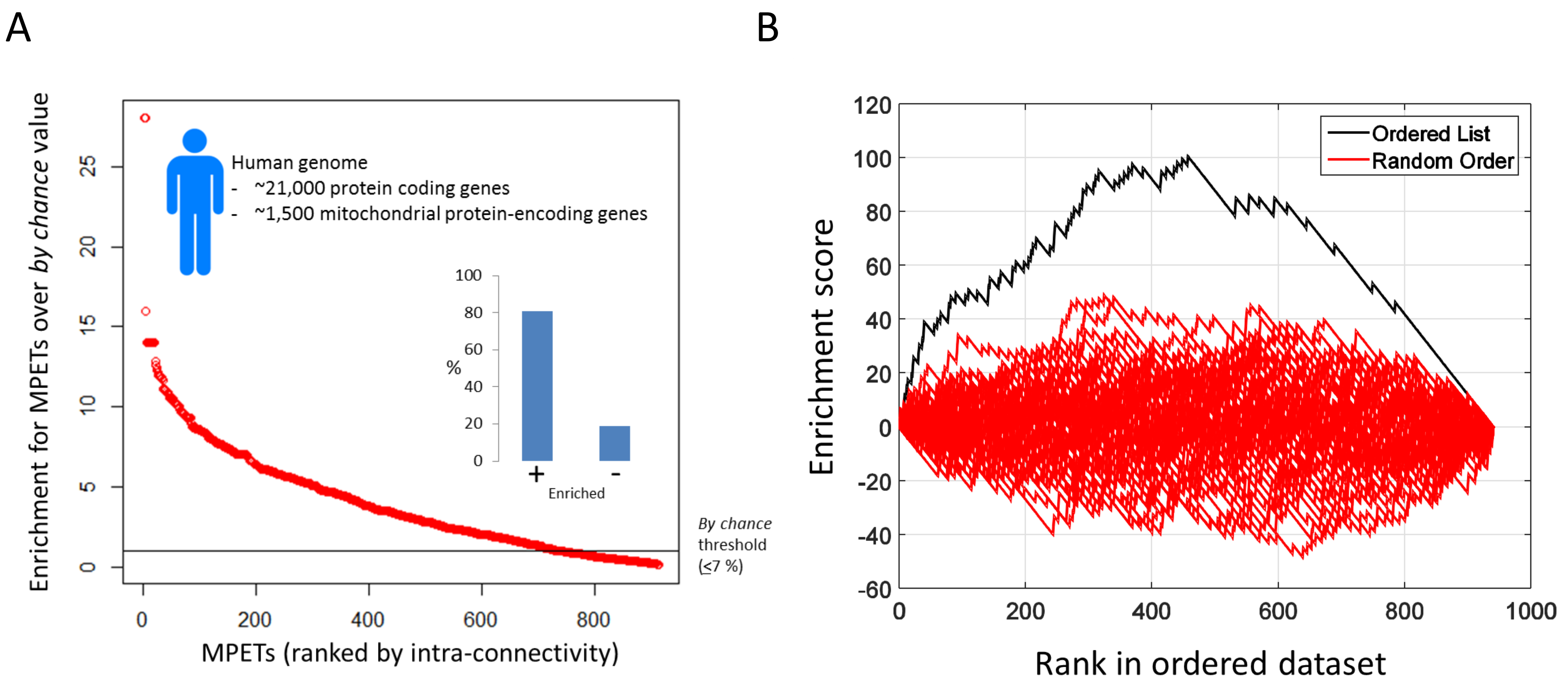

Supplementary Figure 2. Intra-correlation of mitochondrial network seed genes and enrichment of ribosomal protein-encoding transcripts. A, 7% (1,500 of 21,000) of the human genome comprises mitochondrial protein-encoding genes. Hence, a randomly generated gene list will not contain more than 7% mitochondrial protein-encoding genes. The scatter plot (each circle represents a seed gene) reveals that the vast majority (>80%) of the seed genes used to create the mitochondrial network (Figure 1) are co-expressed (R>0.5) with more than 7% mitochondrial protein-encoding genes. Horizontal line displays the 7% *by chance* threshold. Inset bar chart displays the percentage of seeds that correlate with >7% (+) or ≤7% (-) MPETs. B, Distribution of transcripts known to generate mitochondrial ribosomal proteins was analyzed via enrichment analysis. On descending the ranked list (in which transcripts are ranked by the number of times they correlate at $R \geq 0.5$ with other seed transcripts), up- and down-step scores were applied to transcripts producing ribosomal proteins and those not, respectively (see Methods). The maximum enrichment score (100) was significantly greater than that obtained from 1000 random walks (shown in red).
