# Supplementary material for: Transcriptome-wide co-expression analysis identifies LRRC2 as a novel mediator of mitochondrial and cardiac function
Source: PLoS One. 2017 Feb 3;12(2):e0170458. doi: 10.1371/journal.pone.0170458 (PMC5291451; doi:10.1371/journal.pone.0170458)
Supplement: S3 Fig — A, Percentage of transcripts within and outside the LE that generate proteins reported (in the BIOGRID database) to interact with ≥1, ≥2, ≥5, and ≥10 known mitochondrial proteins. B, Relative abundance of MC- proteins (proteins that are not annotated by MitoCarta as being mitochondrially-loacalized but which are reported (in the BIOGRID database) to interact with known mitochondrial proteins) that interact with ≥1, ≥2, ≥5, and ≥10 known mitochondrial proteins in the LE compared to outside the LE. (PDF) [file pone.0170458.s003.pdf]

Supplementary Figure 3

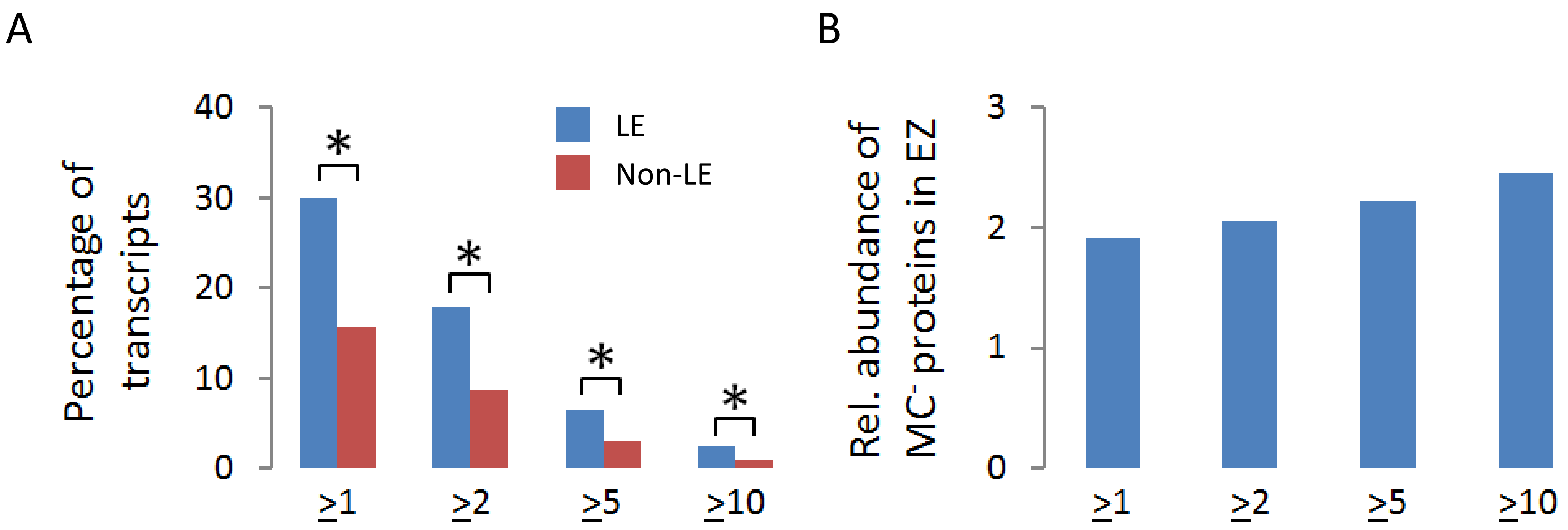

Supplementary Figure 3. Prioritized transcript population is enriched for protein products that physically interact with known mitochondrial proteins. A, Percentage of transcripts within and outside the LE that generate proteins reported (in the BIOGRID database) to interact with ≥1, ≥2, ≥5, and ≥10 known mitochondrial proteins. B, Relative abundance of MC^- proteins (proteins that are not annotated by MitoCarta as being mitochondrially-loacalized but which are reported (in the BIOGRID database) to interact with known mitochondrial proteins) that interact with ≥1, ≥2, ≥5, and ≥10 known mitochondrial proteins in the LE compared to outside the LE.
