# Supplementary material for: Transcriptome-wide co-expression analysis identifies LRRC2 as a novel mediator of mitochondrial and cardiac function
Source: PLoS One. 2017 Feb 3;12(2):e0170458. doi: 10.1371/journal.pone.0170458 (PMC5291451; doi:10.1371/journal.pone.0170458)
Supplement: S4 Fig — Networks were constructed using the three sub-groups (control samples only, idiopathic samples only and ischemic samples only) as well as all samples simultaneously. In each case, horizontal lines (branches) represent modules and vertical lines (leaves) represent transcripts. Asterisks and accompanying P values denote MPET-enriched modules and Benjamini-corrected P values obtained via DAVID-based GO term analysis, respectively. (PDF) [file pone.0170458.s004.pdf]

Supplementary Figure 4

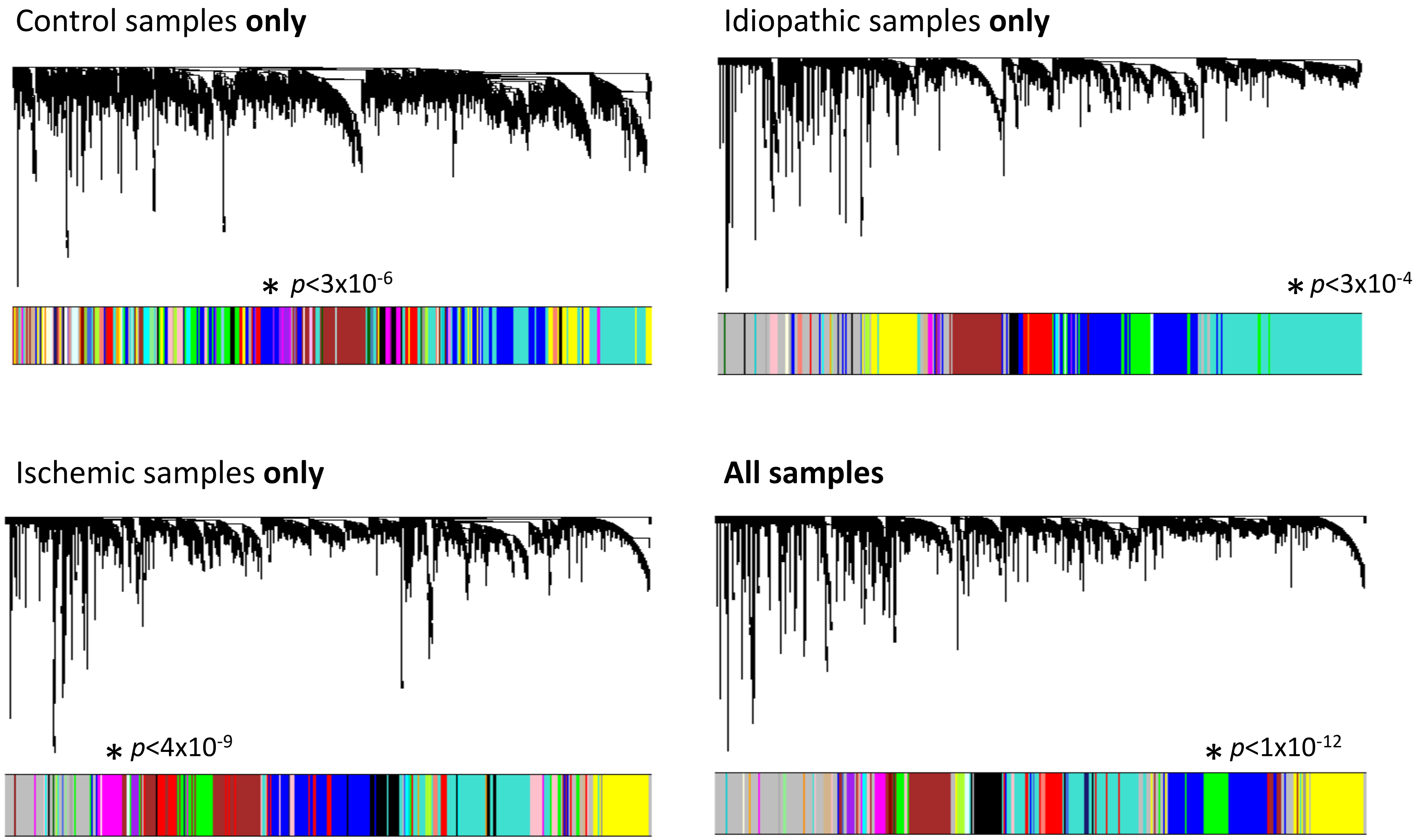

Supplementary Figure 4. Weighted gene co-expression network analysis (WGCNA) of individual sample groups from dataset GSE5406. Networks were constructed using the three sub-groups (control samples only, idiopathic samples only and ischemic samples only) as well as all samples simultaneously. In each case, horizontal lines (branches) represent modules and vertical lines (leaves) represent transcripts. Asterisks and accompanying *P* values denote MPET-enriched modules and Benjamini-corrected *P* values obtained via DAVID-based GO term analysis, respectively.
