# Supplementary material for: Transcriptome-wide co-expression analysis identifies LRRC2 as a novel mediator of mitochondrial and cardiac function
Source: PLoS One. 2017 Feb 3;12(2):e0170458. doi: 10.1371/journal.pone.0170458 (PMC5291451; doi:10.1371/journal.pone.0170458)
Supplement: S5 Fig — H9c2 cells transfected with either a control or LRRC2-specific siRNA were stained with Mitotracker Green and DAPI before being imaged via confocal microscopy (x100). Representative images demonstrate no overt alteration in the morphology of the mitochondrial network. Scale bar = 20μm. (PDF) [file pone.0170458.s005.pdf]

Supplementary Figure 5

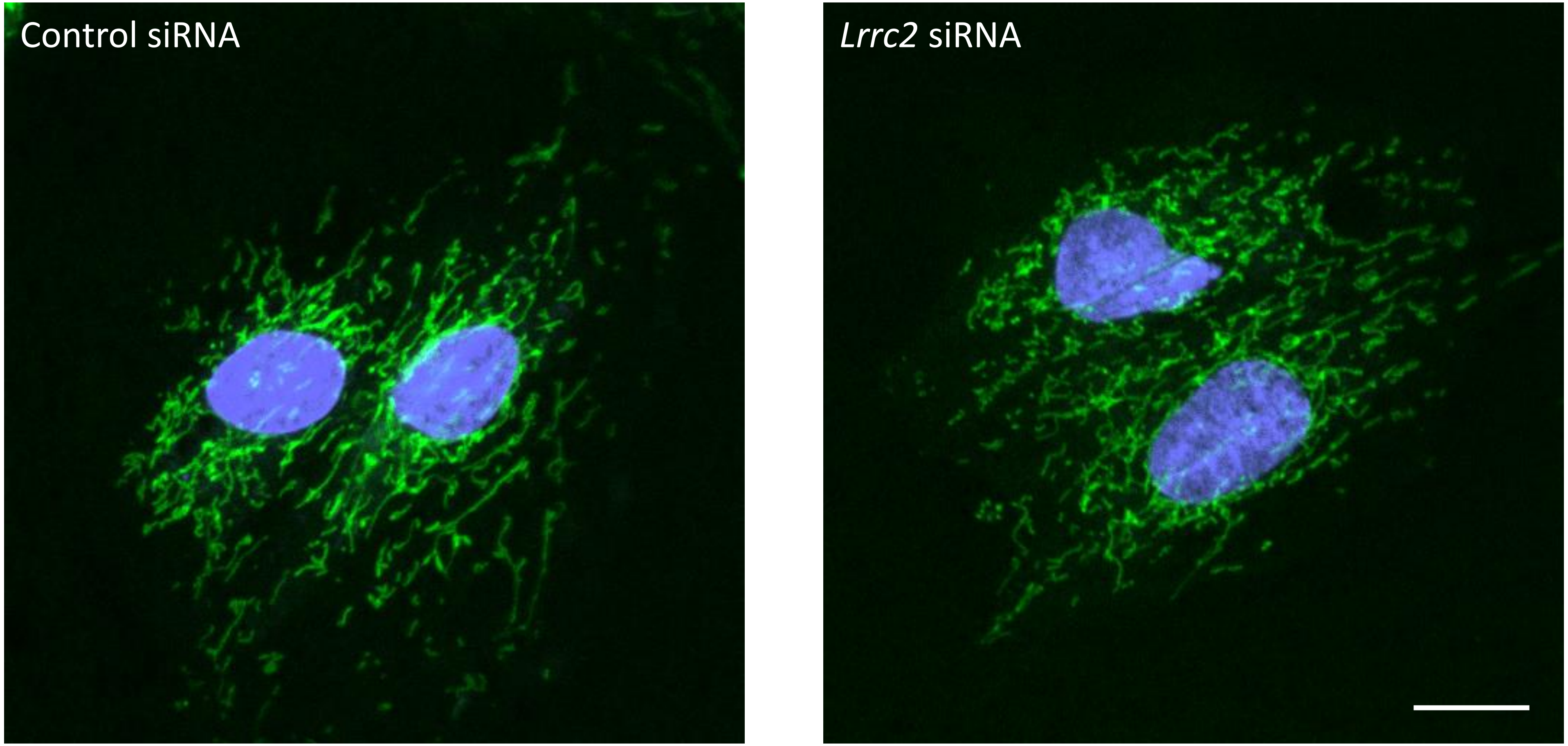

Supplementary Figure 5. Mitochondrial morphology is not altered by LRRC2 loss-of-function. H9c2 cells transfected with either a control or *LRRC2*-specific siRNA were stained with Mitotracker Green and DAPI before being imaged via confocal microscopy (x100). Representative images demonstrate no overt alteration in the morphology of the mitochondrial network. Scale bar=20µm.
