# Supplementary material for: Transcriptome-wide co-expression analysis identifies LRRC2 as a novel mediator of mitochondrial and cardiac function
Source: PLoS One. 2017 Feb 3;12(2):e0170458. doi: 10.1371/journal.pone.0170458 (PMC5291451; doi:10.1371/journal.pone.0170458)
Supplement: S6 Fig — H9c2 cells were infected with either a GFP adenovirus alone (1st bar), equal amounts of a GFP and Pgc-1α adenovirus (2nd bar), equal amounts of a Pgc-1α and a LRRC2 adenovirus (3rd column), or equal amounts of a GFP and LRRC2 adenovirus (4th bar). RNA was isolated and subjected to QPCR with Pgc-1α-specific primers capable of detecting transcript derived from the adenovirus and the endogenous gene (and HPRT primers for normalization). Data are represented as mean ± s.e.m from three independent experiments. *, P<0.05. (PDF) [file pone.0170458.s006.pdf]

Supplementary Figure 6

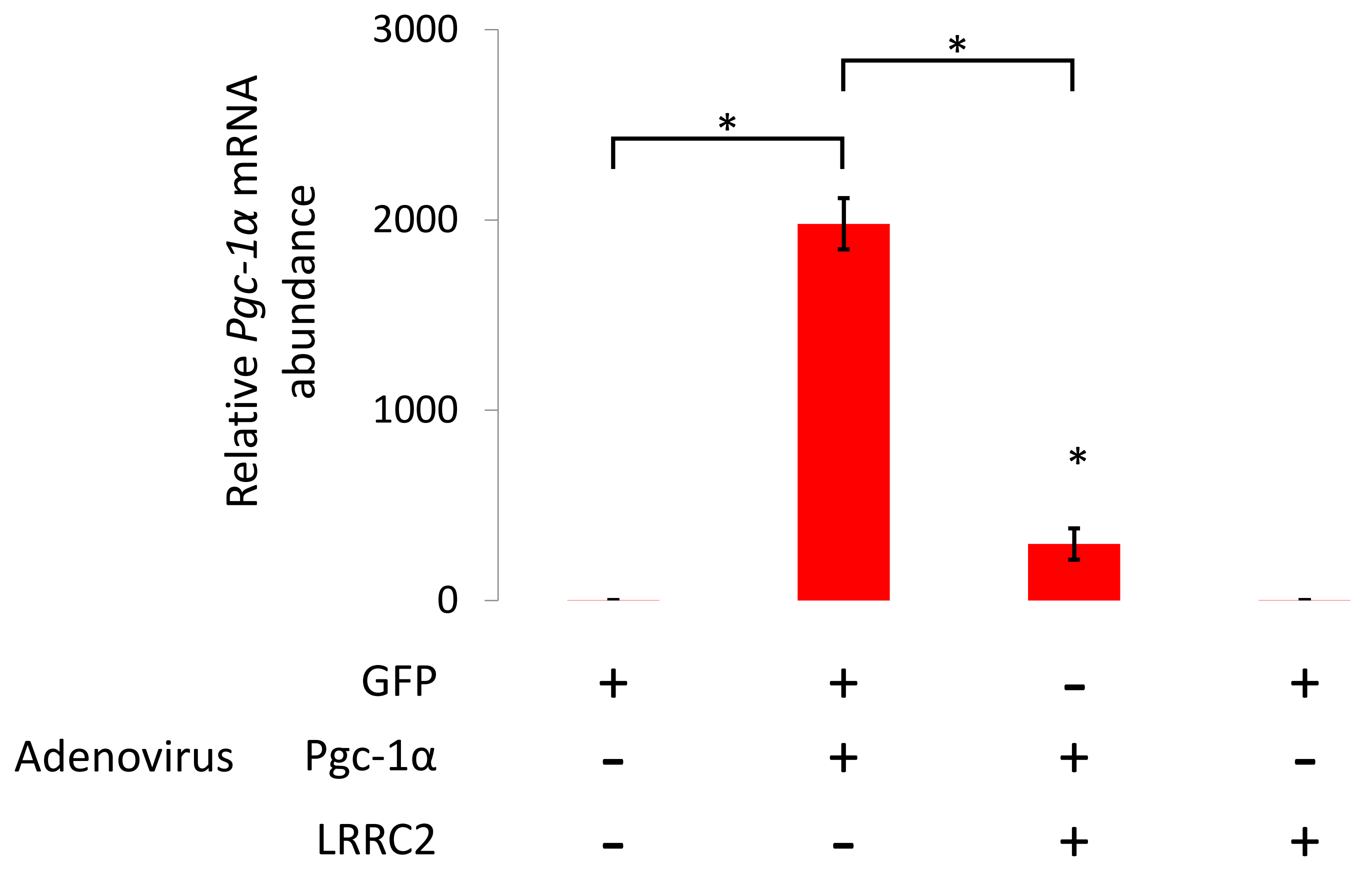

Supplementary Figure 6. LRRC2-mediated repression of *Pgc-1α* induction in H9c2 cardiomyocytes. H9c2 cells were infected with either a GFP adenovirus alone (1st bar), equal amounts of a GFP and Pgc-1α adenovirus (2nd bar), equal amounts of a Pgc-1α and a LRRC2 adenovirus (3rd column), or equal amounts of a GFP and LRRC2 adenovirus (4th bar). RNA was isolated and subjected to QPCR with Pgc-1α-specific primers capable of detecting transcript derived from the adenovirus and the endogenous gene (and HPRT primers for normalization). Data are represented as mean ± s.e.m from three independent experiments. *, $P < 0.05$.
